# Simultaneous Li-Doping and Formation of SnO_2_-Based Composites with TiO_2_: Applications for Perovskite Solar Cells

**DOI:** 10.3390/ma17102339

**Published:** 2024-05-14

**Authors:** Nagisa Hattori, Kazuhiro Manseki, Yuto Hibi, Naohide Nagaya, Norimitsu Yoshida, Takashi Sugiura, Saeid Vafaei

**Affiliations:** 1 Graduate School of Natural Science and Technology, Gifu University, Yanagido 1-1, Gifu 501-1193, Japan; 2 Mechanical Engineering Department, Bradley University, 1501 West Bradley Avenue, Peoria, IL 61625, USA

**Keywords:** SnO_2_, nanoparticles, solid solution, sintering, solar cells

## Abstract

Tin oxide (SnO_2_) has been recognized as one of the beneficial components in the electron transport layer (ETL) of lead–halide perovskite solar cells (PSCs) due to its high electron mobility. The SnO_2_-based thin film serves for electron extraction and transport in the device, induced by light absorption at the perovskite layer. The focus of this paper is on the heat treatment of a nanoaggregate layer of single-nanometer-scale SnO_2_ particles in combination with another metal-dopant precursor to develop a new process for ETL in PSCs. The combined precursor solution of Li chloride and titanium(IV) isopropoxide (TTIP) was deposited onto the SnO_2_ layer. We varied the heat treatment conditions of the spin-coated films comprising double layers, i.e., an Li/TTIP precursor layer and SnO_2_ nanoparticle layer, to understand the effects of nanoparticle interconnection via sintering and the mixing ratio of the Li-dopant on the photovoltaic performance. X-ray diffraction (XRD) and high-resolution transmission electron microscopy (HR-TEM) measurements of the sintered nanoparticles suggested that an Li-doped solid solution of SnO_2_ with a small amount of TiO_2_ nanoparticles formed via heating. Interestingly, the bandgap of the Li-doped ETL samples was estimated to be 3.45 eV, indicating a narrower bandgap as compared to that of pure SnO_2_. This observation also supported the formation of an SnO_2_/TiO_2_ solid solution in the ETL. The utilization of such a nanoparticulate SnO_2_ film in combination with an Li/TTIP precursor could offer a new approach as an alternative to conventional SnO_2_ electron transport layers for optimizing the performance of lead–halide perovskite solar cells.

## 1. Introduction

In recent years, perovskite solar cells (PSCs) have attracted much attention as a new renewable energy technology [1,2,3,4,5]. They are characterized by a low-cost production process, such as solution deposition, including various printing techniques. This allows for the production of PSCs with fewer installation constraints. Since the initial report on PSCs was published by Miyasaka et al. in 2009 employing the device structure of dye-sensitized solar cells [6], there has been rapid technological innovation in a short period, driven by advancements in variable materials and device structures. Currently, the highest photovoltaic conversion efficiency has reached 26%, rivaling the silicon-based solar cells that are widely used today [7]. The improvement of charge separation and transport efficiencies has been examined by diverse studies employing various electron and hole transport materials. The n-i-p and inverted p-i-n-type device architectures have been developed [8].

The key factor contributing to the improved conversion efficiency of PSCs is the high open-circuit voltage (V_oc_). The elevated V_oc_ exceeding 1V in organic lead iodide semiconductors, unlike in most dye-sensitized solar cells, arises from minimal voltage loss from the bandgap of perovskite compounds. This characteristic property can suppress thermal losses due to the charge recombination of electrons and holes. In high-efficiency devices utilizing lead–halide perovskite compounds, direct recombination involving deep traps within the semiconductor bandgap is minimal, while recombination involving shallow traps is considered to dominate the photovoltaic properties. The recombination associated with these shallow traps is mainly influenced by defects occurring at the interfaces between the perovskite crystals and the charge transport layer. Typically, the carrier mobility in the perovskites allows for long diffusion lengths of over 1 μm for electrons and holes. However, recombination related to the mentioned shallow traps is considered to reduce the diffusion length of photoexcited carriers, leading to voltage losses. In other words, suppressing recombination through materials design of charge transport layers contributes to an increase in photovoltaic conversion efficiency [9,10,11,12,13,14].

The electron transport layer of high-performance n-i-p PSCs often utilizes titanium dioxide (TiO_2_) and tin oxide (SnO_2_), whereas organic fullerene-based compounds, such as PCBM, were widely used as an electron transport layer in inverted PSCs. For n-i-p PSCs, one advantage of SnO_2_ is its high electron mobility [15]. SnO_2_ exhibits a significantly high carrier mobility in the range of 100 to 200 cm^2^V⁻^1^s⁻^1^. Notably, a synergistic effect has been discovered in improving the power conversion efficiency by combining SnO_2_ with TiO_2_ [16,17,18,19,20,21,22]. One of the strategies for the electron transport materials includes creating a cascading electron transfer path from the relatively high-conduction band of TiO_2_ to SnO_2_. Additionally, if an SnO_2_ film surface is covered with a TiO_2_ layer, it is anticipated that the electron transport layer’s performance will be enhanced, leading to an improvement in the interface contact with the perovskite layer.

In addition, numerous reports have been reported on PSCs employing metal-doped SnO_2_ materials for electron transport. One of the major advantages was found in the Li-doped electron transport layer, which was prepared by a low-temperature solution process [23]. The downward shift of the conduction band as well as the conductivity enhancement allowed for efficient electron injection and transport and reduced charge recombination. The effects of Li-doping in TiO_2_ have also been developed by several groups in their TiO_2_-based PSCs (using TiO_2_ solely as an electron transport layer). Li doping has been shown to improve the crystallinity of titanium dioxide and consequently enhance its performance. Additionally, partial reduction of tetravalent titanium to trivalent titanium by doping lithium promotes the passivation of electron defects in the TiO_2_, leading to performance improvements [24,25].

However, the impact of SnO_2_, especially with single-nanometer-scale structures, on the process of metal-doping (using post-heating) remains unclear. Moreover, the concept of the creation of nanoparticulate SnO_2_-based solid-solutions with TiO_2_ has not yet been applied to the metal-doped ETL in PSCs.

In our previous paper [26], we demonstrated the use of phenylalanine methyl ester hydrochloride in the solution synthesis of SnO_2_ nanoaggregates. This allowed us to tune primarily particle sizes to be as small as 3 nm while enhancing the SnO_2_ crystallinity with the assistance of the hydrochloride salt that contributed to promoting the hydrolysis of Sn(IV) sources. In a preliminary experiment, we also showed a spin-coat method to produce a double-layered thin film precursor consisting of the obtained SnO_2_ nanocrystals synthesized at different temperatures (deposited at the bottom) and a TTIP coating layer (deposited on the bottom layer), which were used for the fabrication of PSCs.

Using the SnO_2_ nanoaggregates, we report on the first simultaneous Li-doping and the formation of an SnO_2_ nanoaggregate-based solid solution with a small amount of TiO_2_ nanoparticles. A narrower bandgap as a result of the formation of SnO_2_/TiO_2_ solid solution was also proved as opposed to that of SnO_2_. We varied the sintering conditions and the mixing ratio of the Li-dopant for the Ti precursor solution to gain insight into the ETL performance of the Li-doped solid solution in PSCs.

## 2. Materials and Methods

### 2.1. Chemicals

L-phenylalanine methyl ester hydrochloride and tin(IV) chloride dihydrate (SnCl_4_·5H_2_O, >98.0%) were obtained from FUJIFILM Wako Pure Chemical Corporation, Osaka, Japan. Titanium isopropoxide (TTIP, >97%) and lithium chloride (LiCl, 99.0%) were purchased from KANTO CHEMICAL Co., Inc., Tokyo, Japan.

Dehydrated solvents, N,N-dimethylformamide (DMF, >99.5%), dimethyl sulfoxide (DMSO, 99.0%), 2-propanol (IPA > 99.7%), and acetonitrile (CH_3_CN > 99.5%) were supplied from KANTO CHEMICAL Co., Inc., Tokyo, Japan. Ethanol (99.5%) was from Kanto Chemical Co., Inc., Tokyo, Japan. A Milli-Q^®^ integral water purification system (MERCK Ltd., Tokyo, Japan) was used for obtaining H_2_O (resistivity: 18.2 MΩ·cm). All the chemicals were used as purchased for the experiments.

For the fabrication of perovskite solar cells, lead(II) iodide (PbI_2_, 99.99%), methylamine hydrobromide (MABr, >98.0%), cesium iodide (CsI, >99.0%), formamidine hydroiodide (FAI, 99.99%), lithium bis(trifluoromethanesulfonly)imide (Li-TFSI, >98.0%), and formamidine hydrobromide (FABr, 99.99%) were obtained from TOKYO CHEMICAL INDUSTRY CO., Ltd., Tokyo, Japan. Spiro-MeOTAD, chlorobenzene (CB, 99.8%), and 4-tert-butylpyridine (TBP, 98%) were from SIGMA-ALDRICH, Co., St. Louis, MO, USA.

### 2.2. Preparation of Li-Doped SnO_2_ Composites with TiO_2_

SnO_2_ nanoaggregates were synthesized as follows [26]. After dissolving L-phenylalanine methyl ester hydrochloride (0.43 g) in a mixed solvent of 20 mL of ethanol and water (ethanol:water = 1:1 *v*/*v*), tin(II) chloride pentahydrate (0.71 g) was added. The solution was stirred at 80 °C for 48 h. After drying the resultant solution in air, 10 mL of ethanol was added to dissolve the mixture. The solution was then spin-coated onto a fluorine-doped tin oxide (FTO) substrate at 3500 RPM for 30 s and then heated at 120 °C for 5 min.

**Figure 1:**
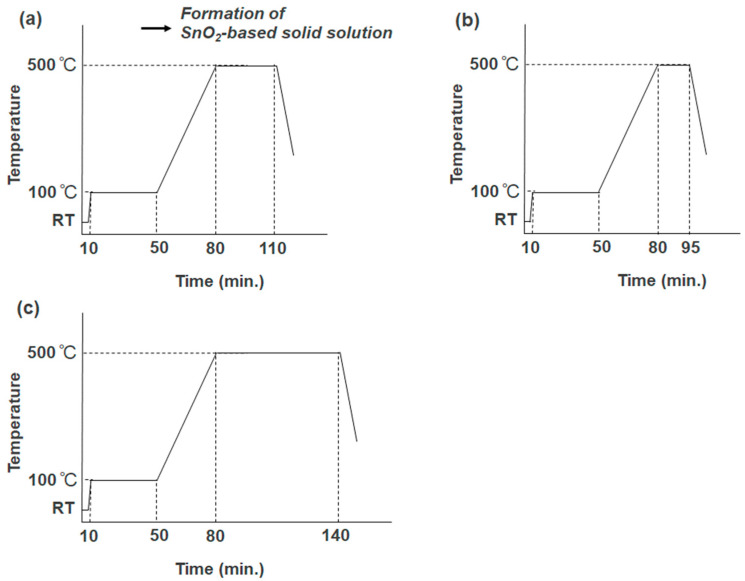
Sintering strategies of SnO_2_-based solid solution with TiO_2_. The hold times at the maximum temerature of 500 °C were (**a**) 30 min, (**b**) 15 min, and (**c**) 1 h, respectively.

A precursor solution was prepared by mixing ethanol, water, nitric acid, and TTIP. In the case of Li-doping, lithium chloride was dissolved in this solution (Ti:Li = 100:1, 20:1 (mol/mol)). The prepared solution was spin-coated onto the SnO_2_ layer in a two-step process (step 1: 1500 RPM, 30 s, and step 2: 1000 RPM, 60 s). Based on a method previously published by our group [27], it was subsequently heated at 500 °C after preheating at 100 °C in an electric furnace, KDF 300 Plus (DENKEN-HIGHDENTAL, Kyoto, Japan). The strategies for sintering nanoparticles are presented in Figure 1, where the duration times at 500 °C were varied.

The methodology of nanoparticle deposition and subsequent sintering significantly influences the electrical conductivity of the semiconductor nanoparticle layer [28]. For nanoparticle deposition, optimizing the rotating angular velocity (RPM) and deposition duration is crucial to achieving a homogeneous and appropriate thickness of the nano-layer. Similarly, in the case of sintering, the ramping rate, duration at maximum temperature, and the maximum temperature itself play significant roles in determining the porosity of the deposited nanoparticles [29]. Therefore, these parameters need to be optimized to maximize electrical conductivity or electron mobility. As the porosity of deposited semiconductor nanoparticles increases, electrical conductivity decreases, while the interfacial area between semiconductor nanoparticles and light-absorbing materials increases. Hence, porosity must be optimized to enhance the electron mobility of deposited nanoparticles.

In our study, we set the maximum sintering temperature for nanoparticles at 500 °C due to temperature constraints imposed by FTO glasses. Additionally, we varied the duration of sintering to optimize the performance of the solar cell.

### 2.3. Structure Analysis of SnO_2_/TiO_2_ Solid-Solution and Their ETL Films

SnO_2_-based solid solution nanoparticles were characterized using X-ray diffraction (XRD, Rigaku RINT Ultima/PC with monochromated Cu–Kα radiation, Tokyo, Japan). The analysis of the nanostructures was performed by means of TEM (JEM-2100, JEOL, Tokyo, Japan). The diffuse reflectance spectra of the nanoparticles were obtained using a UV–Vis spectrophotometer (V-770, Jasco, Tokyo, Japan). A surface and cross-sectional image of ETL/FTO substrates were obtained using SEM (S-4800, Hitachi High-Tech Corporation, Tokyo, Japan).

### 2.4. Fabrication of Perovskite Solar Cells

We deposited the perovskite layer onto the ETL using a modified process previously reported [30]. All procedures were performed within a glove box filled with nitrogen at a relative humidity of 20%. Prior to the deposition of the perovskite precursor solution, the ETL/FTO substrate was heated to 80 °C. The perovskite solution was prepared as follows: CsI (0.07 mmol), FAI (1.19 mmol), MABr (0.14 mmol), and PbI_2_ (1.45 mmol) were dissolved in a mixture of 80 vol% DMF (0.8 mL) and 20 vol% DMSO (0.2 mL). The mixture was stirred for 2 h at 70 °C on a hot plate. The substrate was then placed in a spin-coater, and the perovskite solution was dropped onto the substrate with a two-step coating process (1000 RPM for 10 s, followed by 6000 RPM for 30 s). After spin-coating, 300 μL of CB was added. The substrate was further annealed at 100 °C for 1 h and then cooled naturally to room temperature. In parallel, FABr (0.12 mmol) was added to 4 mL of IPA to prepare a solution. The FABr solution was coated onto the perovskite layer at 4000 RPM for 20 s, and the substrate was heated at 80 °C for 10 min. After heating, the substrate was cooled to room temperature.

A total of 0.045 g of Spiro-MeOTAD was dissolved in 0.5 mL of CB. Separately, 0.052 g of LiTFSI was dissolved in 0.1 mL of acetonitrile. Then, 10 μL of the CH_3_CN solution and 17.75 μL of TBP were added to the Spiro-MeOTAD solution. The prepared solution was then spin-coated onto the perovskite layer at 4000 rpm for 20 s. The substrate was removed from the glove box and left to stand in air for 24 h. Finally, a gold counter electrode was deposited using vacuum evaporation, following the method reported by our group [31]. One-step gold deposition was performed at a pressure of 2 × 10^−3^ Pa, with the gold layer being deposited by adjusting the source power of the thermal evaporation deposition machine.

### 2.5. Evaluation of Solar Cell Performance

The I–V curves were measured under simulated sunlight (100 mW/cm^2^) using a solar simulator (YSS-80A, Yamashita Denso, Tokyo, Japan) with a potentiostat (HSV-110, Meiden Hokuto, Tokyo, Japan). The active area of the PSCs was 0.09 cm^2^. Scanning conditions for the I–V measurements were at a scan rate of 50 mV/s. A Desktop Spectral Response and IPCE Measurement System (Bunkokeiki SM-250, Tokyo, Japan) was used for the measurement of a spectrum of monochromatic incident photon-to-current conversion efficiency (IPCE).

## 3. Results and Discussions

**Figure 2:**
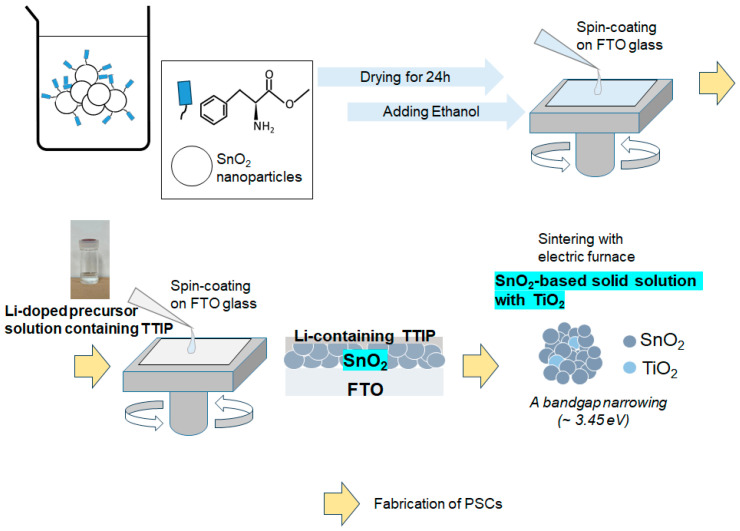
Schematics of the formation of SnO_2_-based solid solution with TiO_2_ as an electron transport layer in perovskite solar cells. SnO_2_ nanoaggregates were synthesized using our structure directing agent (SDA)-supported hydrolysis method, previously reported in [26]. In the final step of ETL preparation, sintering at 500 °C produced the Li-doping SnO_2_-based solid solution with TiO_2_, followed by the deposition of the lead–halide perovskite layer on top of the ETL. Annealing steps (drying process) after the two spin-coating were omitted for clarity. A bandgap narrowing of SnO_2_ nanoparticles induced by TiO_2_ nanoparticles was observed.

One of the important steps in preparing ETLs for PSCs involves the sintering process of oxide nanoparticles. The focus should be on how the resultant materials are grown from a precursor solution through conversion via heat treatment. The successive spin-coating and sintering processes of nanoparticles, along with the preparation of the precursor solution, are illustrated in Figure 2. Additionally, the figure presents that an SnO_2_-based solid solution with TiO_2_ can be formed. Moreover, we anticipate that the additional process of depositing an Li-containing titanium(IV) isopropoxide (TTIP) solution on top of a nanostructured SnO_2_ bottom layer could provide a beneficial platform for realizing effective metal doping in the SnO_2_ core during heat treatment. This approach could offer a favorable method for optimizing electron transport in thin films in PSCs.

Structural investigation of sintered oxide nanoparticles in ETLs is crucial for understanding the growth mechanism of the resultant solid solution from the mixed system of SnO_2_ and Ti(IV) sources. However, microstructural analysis, especially of the sintered ETL, has not been well conducted. This was achieved particularly using a high-resolution transmission electron microscope (TEM), as discussed below. Sintering plays a significant role in controlling the crystal growth of nanoparticles. It is important to determine optimized heat-treatment conditions of ETLs, considering changes in several factors such as maximum temperatures, hold times at the temperatures, and ramping rates of heating. We systematically investigated the sintering conditions at 500 °C, while varying the periods of maximum temperature. Additionally, the Li-dopant rate was varied to understand the effects of doping on the photovoltaic properties.

**Figure 3:**
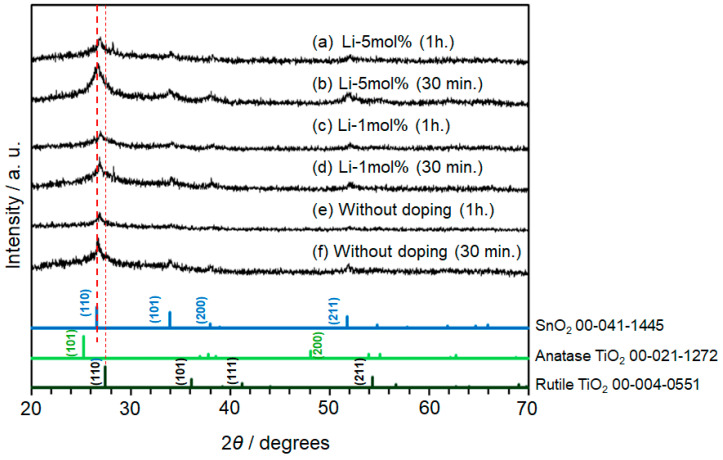
XRD patterns of the sintered samples of SnO_2_-based solid solution with TiO_2_ are shown in (**a**–**d**). Non-doped samples are presented as a control experiment in (**e**,**f**). The database of SnO_2_ and TiO_2_ (anatase and rutile) is also provided at the bottom. For the XRD patterns in (**a**–**d**), the Li-dopant rates were 1 mol% and 5 mol%, and the hold time of heating were 30 min and 1 h. Red dotted lines are shown for clarity.

Figure 3 displays the XRD patterns of the sintered SnO_2_ samples. For Figure 3a–d, the Li-dopant rates for the TTIP solution were 1 mol% and 5 mol%, and hold times during heating at 500 °C were 30 min and 1 h. As a control, non-doped samples are also measured and presented in Figure 3e,f. All observed peaks were assigned to rutile TiO_2_ and SnO_2_ only, indicating that the heating process at 500 °C resulted in the incorporation of TiO_2_ into SnO_2_. No other impurities, such as Li and its oxides, were detected when the doping concentration was increased, suggesting that the TiO_2_ or SnO_2_ crystal lattice were partially replaced by a Li ion with a similar ionic radius and defect formation was suppressed. Regarding hold times at maximum temperature during heating, cracks on the TiO_2_ or SnO_2_ surface increased with increasing time.

Notably, the peak intensity increased with increasing Li concentration in the precursor solution for Li-5 mol% samples, leading to improved crystallinity. Using the Scherrer equation, the crystallite size in each sample was calculated as shown in Table 1. In fact, the calculated crystallite sizes increased when the doping rate was high, while the opposite trend was observed for samples sintered for 1 h. The results suggest that the hold time during sintering is a decisive factor in adjusting the growth of nanoparticles.

**Figure 4:**
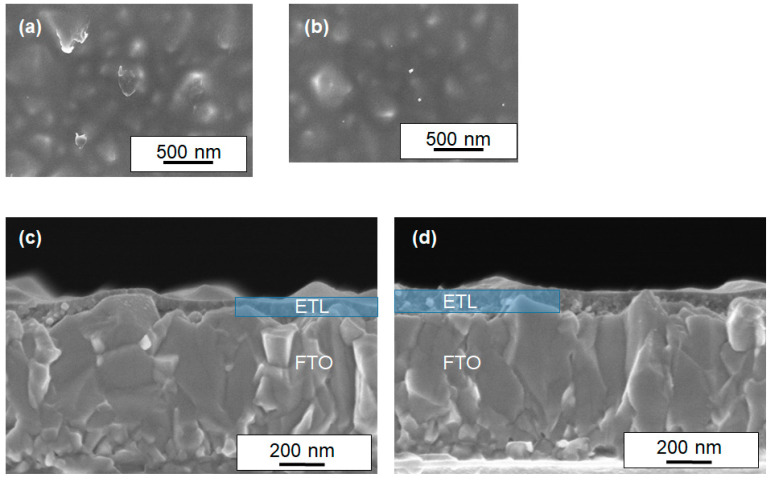
SEM images of the sintered samples of SnO_2_-based solid solution with TiO_2_ are provided. Non-doped samples (**a**,**c**) and Li-doped (5 mol%, **b**,**d**) samples are presented. Images (**a**,**b**) depict the surface of the films, while (**c**,**d**) show cross-sectional images of the ETL/FTO substrates. The ETL layers are highlighted in blue.

To investigate the influence of Li doping on the morphology of ETLs, we carried out the scanning electron microscope (SEM) to observe the undoped and Li-doped films. As shown in Figure 4, these SEM images show that the morphologies of the films were not affected by Li doping. It was also observed that a dense and smooth layer without pinholes was formed. Cross-sectional SEM images showed that the thickness of both thin films was about 50–100 nm, with particle sizes ranging from 10 to 30 nm. The film thickness is very thin, and the surface SEM image shows some uneven structure of the FTO, which is thought to indicate that the surface of the FTO is not completely covered.

**Figure 5:**
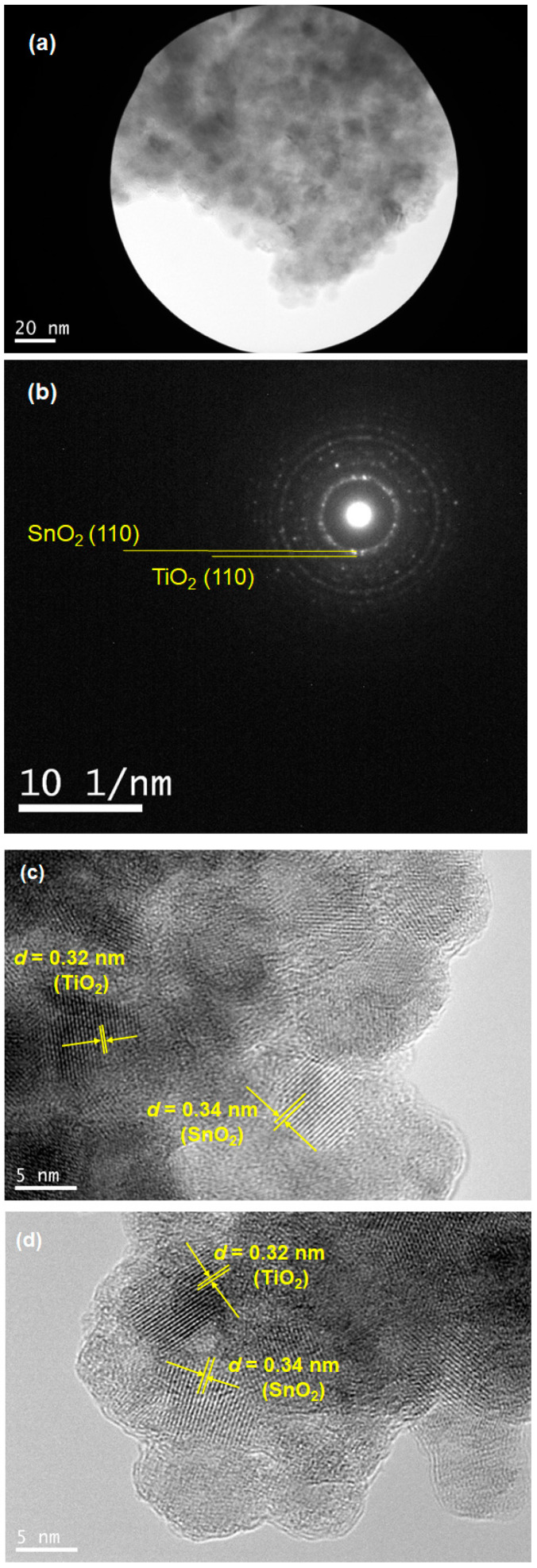
TEM images of the SnO_2_-based solid solution with TiO_2_ (Li-5 mol%) are presented. Images (**a**,**b**) show the sintered nanoparticle and its corresponding selected area diffraction (SAD) pattern (scale bar in (**b**): 10 nm^−1^), respectively. Images (**c**,**d**) are high-resolution images observed at different positions. D-spacings of both TiO_2_ and SnO_2_ for (**c**,**d**) are also presented.

TEM measurements provide a straightforward insight into the formation of the SnO_2_-based solid solution. Figure 5 shows TEM images of an SnO_2_-based solid solution with TiO_2_ (Li-5 mol%, 30 min sintering). Figure 5a,b depicts the bright-field image of the sintered nanoparticles and the selected area diffraction (SAD) pattern of the same position, respectively. It turned out that the diffraction spots of both TiO_2_ and SnO_2_ were of (110) crystal planes. Along with the result, high-resolution images of (Figure 5c,d) clearly indicated the formation of rutile TiO_2_ and SnO_2_ nanoparticles, with approximate crystal sizes of 5 nm. The dimensions of the crystals were in agreement with the calculated data shown in Table 1. The estimated *d*-spacings for the observed nanoparticles were 0.32 nm for TiO_2_ and 0.34 nm for SnO_2_, which are consistent with the SAD pattern.

**Figure 6:**
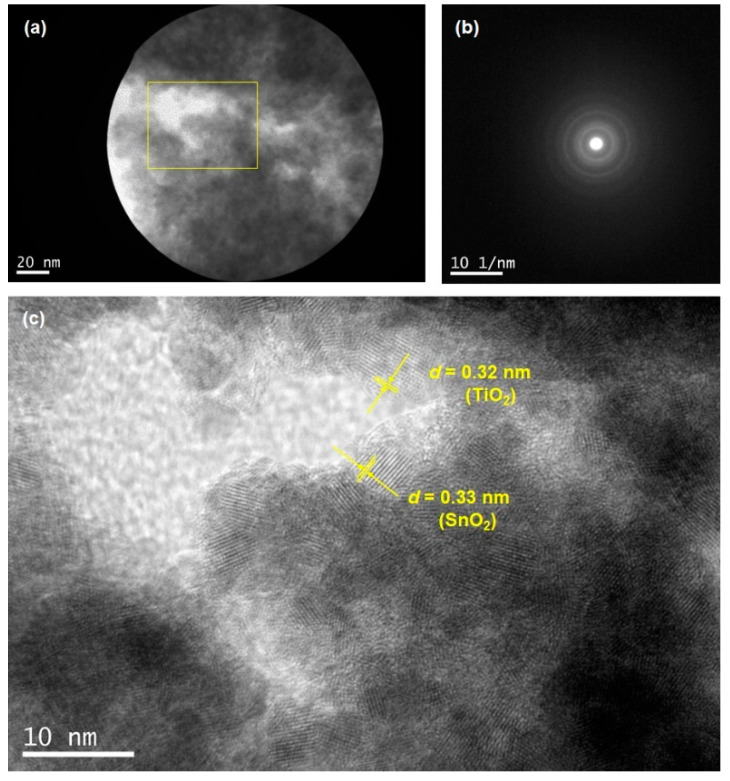
TEM images of the SnO_2_-based solid solution with TiO_2_ (Li-1 mol%) are provided. Images (**a**,**b**) display the sintered nanoparticle and its corresponding SAD pattern (scale bar in (**b**): 10 nm^−1^), respectively. Image (**c**) is a high-resolution image corresponding to the location highlighted in yellow in image (**a**). D-spacings of both TiO_2_ and SnO_2_ are also presented.

Figure 6 also presents data for an SnO_2_-based solid solution with TiO_2_ (Li-1 mol%, 30 min sintering). The SAD ring pattern was also observed, as shown in Figure 6b. The location highlighted in yellow in Figure 6a corresponds to the high-resolution image of Figure 6c. D-spacings of the lattice fringes were assigned to be 0.32 nm for TiO_2_ and 0.33 nm for SnO_2_, similar to those of the Li-5 mol% data. It was also confirmed that the observed crystal sizes, as small as 5 nm, were consistent with the calculated data. As a result of the heating process at 500 °C, the TEM images in Figure 5 and Figure 6 clearly indicate that nanoparticles were densely packed to form a solid solution of SnO_2_/TiO_2_ due to sintering effects.

**Figure 7:**
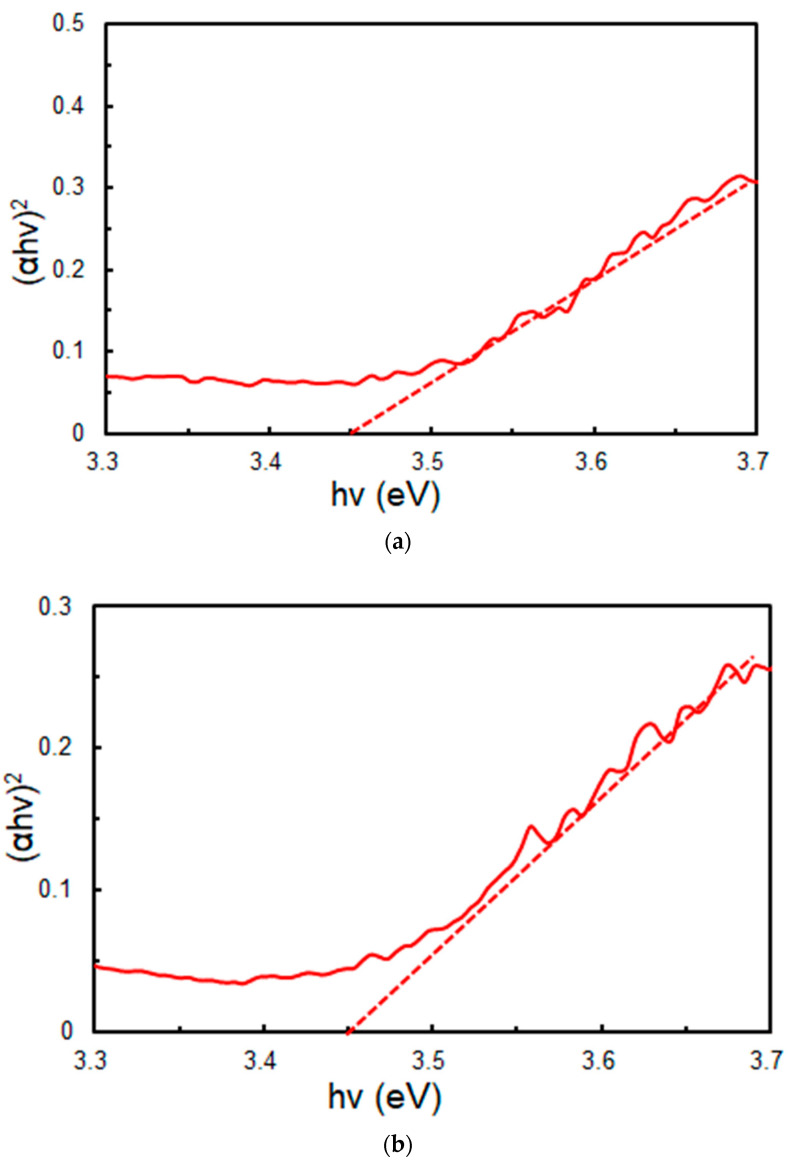
Bandgap estimation of Li-doped SnO_2_-based solid solution ETLs (red solid lines): (**a**) 1 mol% (**b**) 5 mol%. The linear parts of these Tauc plot are extrapolated to the *X*-axis using red dotted lines. The ETLs were sintered at 500 °C for 30 min.

The bandgap of the Li-doped samples (Li-1 mol% and 5 mol%) was estimated to be 3.45 eV for both samples from the diffuse reflection spectra, as shown in Figure 7, irrespective of the Li doping concentration. The bandgap of SnO_2_ is known to be 3.6 eV [32]. It should be noted that the bandgap of their Li-doped samples is considered to be narrowed due to the formation of the solid solution of SnO_2_ and TiO_2_.

**Figure 8:**
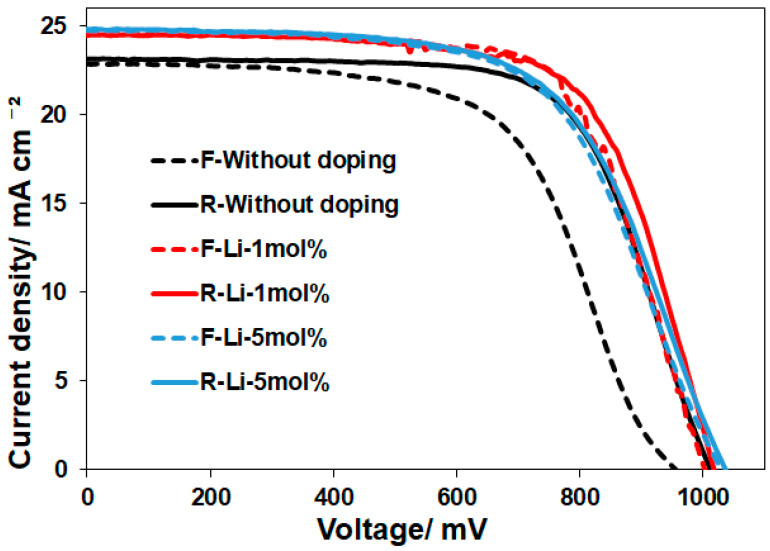
I–V curves of PSCs prepared with the ETLs of Li-doped SnO_2_-based solid solutions. The measurements were conducted under simulated sunlight (100 mW/cm^2^). The data were obtained by reverse (R) and forward scans (F).

To understand the effective Li-doping conditions in ETLs, eighteen different processes were tested to evaluate the performance of the ETLs. Different hold times in the sintering and Li-doping rates were examined. The representative I–V curves for the 30 min sintering were depicted in Figure 8, and all the I–V parameters were summarized in Table 2 and Table 3. Most notably, Li-doping played a significant role in suppressing hysteresis behavior (hysteresis index: HI < 1%), as shown in Table 2. Several groups have reported a similar observation of low HI induced by Li-doping [33]. The hysteresis index was calculated, based on PCE values measured in forward and reverse scans [34]. However, this trend was observed only for the 30 min sintering. It was found that the hold times in the sintering are important factors for determining the photovoltaic performance. There are some reports on Li-doping that can assist in passivating the defects in the TiO_2_ and SnO_2_ films, increasing the conductivity of metal oxides [35,36]. In addition, relatively larger V_oc_ and FF values were observed for the Li-doped samples (Li-1 mol% and 5 mol%) heated for 30 min, leading to an improvement in PCEs. These results can likely be explained by the simultaneous effects of Li-doping and optimized sintering conditions (30 min) of nanoparticles. It is probable that the smaller crystallite sizes of nanoparticles, as shown in Table 1, have an advantage in the enhancement of sintering, allowing for the formation of favored electron transport layers.

**Figure 9:**
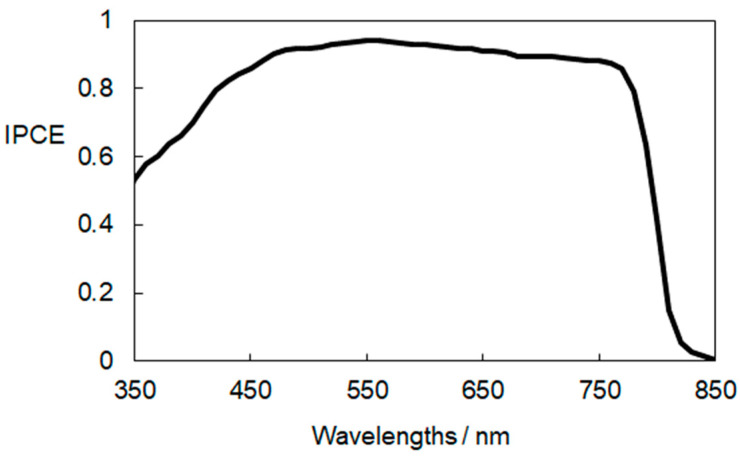
IPCE spectrum of the best-performing PSC with a sintered Li-1 mol% ETL. The hold time at 500 °C in the sintering was 30 min.

To confirm the ability of the perovskite compound to convert light into electrical energy, we conducted measurements of incident photon-to-current conversion efficiency (IPCE). Figure 9 illustrates the IPCE response obtained from a sintered Li-doped ETL (1 mol%)-based PCE, which demonstrated the best performance in this study. The results indicate that a significant portion of visible light and near-infrared radiation, up to approximately 850 nm, can be efficiently converted into electricity. This corresponds to the light absorption characteristics of the lead–halide perovskite compound in the device. Based on the cross-sectional SEM images in Figure 4, it is evident that the partial contact between the perovskite and FTO should be minimized by further adjusting the thickness of the ETLs. This is crucial, as partial contact between the perovskite and FTO can induce charge recombination in the device. Optimization of the device is currently underway by varying the film thickness of the ETLs.

## 4. Conclusions

We have demonstrated that the sintering of the integrated double layers, i.e., a layer of single-nanometer-scale SnO_2_ particles and Li-containing TTIP precursors, enabled the production of Li-doped SnO_2_-based solid solution with TiO_2_, which can be processed with a thickness of 50–100 nm. The combined precursor of lithium chloride and TTIP facilitated effective metal doping via heating in the resultant oxide thin films. Notably, the bandgap of the Li-doped ETL samples was estimated to be 3.45 eV, indicating a narrower bandgap as compared to that of pure SnO_2_ (3.6 eV). Also, hold time in the sintering was found to be a crucial factor determining the performance of PSCs. We varied the heat treatment conditions while changing the mixing ratio of the Li-dopant. The nanostructures of the resultant solid solution materials were successfully characterized using XRD and HR-TEM measurements. Successive spin-coating and sintering that can convert the integrated double layers into a doped SnO_2_-based solid solution as an ETL could offer a beneficial approach as an alternative to conventional SnO_2_ ETLs for high-efficiency lead–halide perovskite solar cells.

## Figures and Tables

**Table 1:** Calculated crystallite sizes of sintered SnO_2_ nanoparticles. The crystallite sizes of the nanoparticles were estimated using the Scherrer equation *.

ETLs	Hold Times in Heating	Calculated Crystallite Sizes (nm)
Without doping	30 min.	2
Without doping	1 h.	11
Li-1mol%	30 min.	2
Li-1mol%	1 h.	7
Li-5mol%	30 min.	4
Li-5mol%	1 h.	5

* D = Kλ/βcos θ, D, K, λ, and θ show the crystallite size, Scherrer constant (0.90), X-ray wavelength (1.54 Å), and Bragg angle, respectively.

**Table 2:** I–V parameters of the PSCs with sintered Li-doped ETLs. All the data were obtained by reverse and forward scans. The hold time at the maximum temperature in the sintering was 30 min. The decreased hysteresis index (HI) of I–V curves induced by Li-doping is also indicated by a red arrow.

	V_oc_ (mV)	J_sc_ (mA/cm^2^)	FF	PCE (%)	HI (%)
Without doping (Reverse: R) (30 min.)	1015	23.1	0.67	15.8	17.1
Without doping (Forward: F) (30 min.)	956	22.9	0.60	13.1	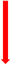
R-Li-1mol% (30 min.)	1027	24.5	0.68	17.0	0.59
F-Li-1mol% (30 min.)	1002	24.6	0.68	16.9	
R-Li-5mol% (30 min.)	1041	24.8	0.62	16.0	1.25
F-Li-5mol% (30 min.)	1030	24.8	0.62	15.8	

**Table 3:** I–V parameters of the PSCs with sintered Li-doped ETLs. The hold time at the maximum temperature in the sintering was 15 min and 1 h. The hysteresis index (HI) of I–V curves is also presented.

	V_oc_ (mV)	J_sc_ (mA/cm^2^)	FF	PCE (%)	HI (%)
R-Without doping (15 min.)	759	23.8	0.63	11.4	4.4
F-Without doping (15 min.)	725	23.8	0.63	10.9	
R-Without doping (1 h.)	995	23.8	0.60	14.2	5.6
F-Without doping (1 h.)	960	23.8	0.57	13.0	
R-Li-1mol% (15 min.)	936	24.8	0.56	12.9	16.3
F-Li-1mol% (15 min.)	887	24.8	0.49	10.8	
R-Li-1mol% (1 h.)	1038	23.3	0.63	15.3	13.1
F-Li-1mol% (1 h.)	980	24.1	0.56	13.3	
R-Li-5mol% (15 min.)	997	25.0	0.55	13.8	16.0
F-Li-5mol% (15 min.)	963	24.8	0.48	11.6	
R-Li-5mol% (1 h.)	1031	24.2	0.62	15.5	5.2
F-Li-5mol% (1 h.)	1002	24.4	0.60	14.7	

## Data Availability

Data are contained within the article.
